# Genome-wide identification of key regulatory lncRNAs in esophageal cancer metastasis

**DOI:** 10.1038/s41392-021-00476-9

**Published:** 2021-02-27

**Authors:** Wen Wen Xu, Can-Can Zheng, Qian Zuo, Jun-Qi Li, Pan Hong, Yan-Ru Qin, Xin-Yuan Guan, Qing-Yu He, Hua-Xin Liao, Bin Li

**Affiliations:** 1 MOE Key Laboratory of Tumor Molecular Biology and Guangdong Provincial Key Laboratory of Bioengineering Medicine, National Engineering Research Center of Genetic Medicine, Institute of Biomedicine, College of Life Science and Technology, Jinan University, Guangzhou, China; 2 MOE Key Laboratory of Tumor Molecular Biology and Key Laboratory of Functional Protein Research of Guangdong Higher Education Institutes, Institute of Life and Health Engineering, College of Life Science and Technology, Jinan University, Guangzhou, China; 3 State Key Laboratory of Esophageal Cancer Prevention and Treatment, Department of Clinical Oncology, First Affiliated Hospital, Zhengzhou University, Zhengzhou, China; 4 Department of Clinical Oncology, Li Ka Shing Faculty of Medicine, The University of Hong Kong, Pokfulam, Hong Kong SAR, China

**Keywords:** Metastasis, Gastrointestinal cancer, Non-coding RNAs, Tumour biomarkers, Drug development

**Dear Editor,**

Metastasis leads to a poor prognosis of patients with esophageal squamous cell carcinoma (ESCC).^1–3^ but the study on cancer metastasis has been hampered by a lack of reliable cell and animal models. Systematic identification and functional validation of metastasis-associated long non-coding RNAs (lncRNAs) and microRNAs (miRNAs), as well as their interactions in ESCC are urgently needed.^4^

To better mimic the progression of metastasis, we performed in vitro and in vivo experiments to select highly invasive and metastatic sublines, designated I6 and LM3, respectively (Supplementary Fig. S1a).^5^ Next-generation sequencing of lncRNAs and miRNAs was carried out and our results suggested that the AC005562.1-hsa-miR-29c regulatory axis was significantly altered in invasive and metastatic cells, and the results were confirmed by qRT-PCR (Fig. 1a, Supplementary Fig. S1b, c, and Supplementary Tables S1, S2). In a tissue microarray consisting of 104 ESCC tissues and 74 normal tissues, AC005562.1 expression was found significantly higher, whereas hsa-miR-29c expression was lower, in the majority of ESCC tissues (Fig. 1b, Supplementary Fig. S1d), which was confirmed by public database (Supplementary Fig. S1e, f). Kaplan-Meier survival analysis indicated that AC005562.1 was negatively associated with the overall survival of ESCC patients (Fig. 1c), whereas high hsa-miR-29c expression was significantly correlated with longer survival (Supplementary Fig. S1g). More importantly, AC005562.1 and hsa-miR-29c expression was found to be associated with tumor invasion and stage (Supplementary Tables S3, S4), and their expression was negatively correlated with each other (Supplementary Fig. S1h). In addition, another tissue microarray consisting of 40 paired primary tumors and metastatic tissues was analyzed, and higher AC005562.1 expression but lower hsa-miR-29c expression was observed in metastatic tumors compared with the corresponding primary tumors (Fig. 1d, Supplementary Fig. S1i).

**Fig. 1:**
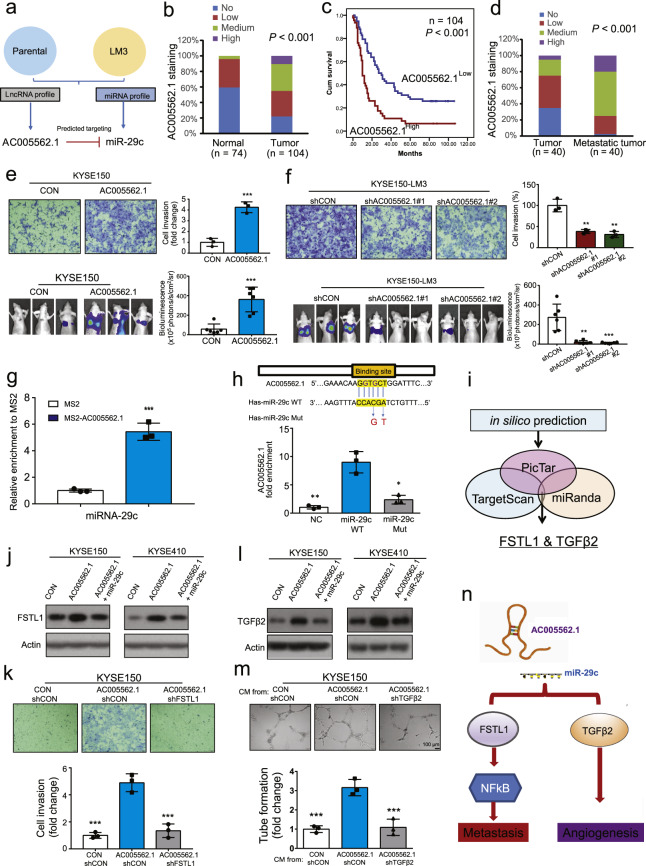
**a** Diagram showing the approaches used in the study to identify the AC005526-hsa-miR-29c regulatory pair. **b** Expression pattern of AC005562.1 and hsa-miR-29c in 74 normal tissues and 104 ESCC tissues. **c** Kaplan-Meier plots were used to compare the overall survival of 104 patients with ESCC stratified according to AC005562.1 or hsa-miR-29c expression. **d** Expression pattern of AC005562.1 and hsa-miR-29c in 40 pairs of primary esophageal cancer and matched metastatic tissues. **e** In vitro and in vivo assay comparing the invasion and metastasis between AC005562.1-overexpressing cells and vector control cells. **f** The invasion and metastasis of AC005562.1-knockdown cells and the vector control cells were compared. **g** MS2 RNA immunoprecipitation showing the binding between hsa-miR-29c and AC005562.1. **h** The wide-type or mutant forms of hsa-miR-29c are shown (upper panel). A pull-down assay showing that AC005562.1 was highly enriched in the sample pulled down by wild-type hsa-miR-29c but not mutant hsa-miR-29c. **i** Diagram showing the strategy to identify direct targets of hsa-miR-29c. **j** Western blot showing the expression of FSTL1 when the AC005562.1 and hsa-miR-29c expression was manipulated. **k** Boyden chamber assay showing the invasion of ESCC cells when the AC005562.1 and FSTL1 expression was manipulated. **l** Western blot showing the expression of TGFβ2 when the AC005562.1 and hsa-miR-29c expression was manipulated. **m** Tube formation assay showing that knockdown of TGFβ2 attenuated the promoting effect of AC005562.1 on angiogenesis in vitro. **n** Summary diagram. Bars, SD; **P < 0.01

Next, we generated stable cell lines overexpressing AC005562.1 or AC005562.1 knockdown (Supplementary Fig. S2a, b), and the results from Boyden chamber invasion and experimental metastasis assays showed that ectopic expression of AC005562.1 increased cell invasion (Fig. 1e and Supplementary Fig. S2c) and the metastasis of ESCC cells to mouse lungs (Fig. 1f and Supplementary Fig. S2d). Western blot showed that AC005562.1 overexpression resulted in the downregulation of E-cadherin and the upregulation of N-cadherin and vimentin (Supplementary Fig. S2e). In contrast, knockdown of AC005562.1 led to a reverse trend in cell invasion (Fig. 1f and Supplementary Fig. 2f), tumor metastasis (Fig. 1f and Supplementary Fig. S2g), and the expression of epithelial and mesenchymal (EMT) markers (Supplementary Fig. S2h). Moreover, neither overexpression nor knockdown of AC005562.1 had an obvious effect on cell proliferation, which excluded the possibility that the invasive-promoting effect of AC005562.1 was due to its proliferation-enhancing ability (Supplementary Fig. S2i). In addition, the suppressive effects of hsa-miR-29c on invasion, metastasis, and EMT phenotypes of ESCC cells were demonstrated in vitro and in vivo (Supplementary Fig. S3).

We next studied the molecular mechanisms underlying the role of AC005562.1 in ESCC. First, our results revealed that AC005562.1 overexpression significantly decreased hsa-miR-29c expression, whereas AC005562.1 knockdown resulted in the upregulation of hsa-miR-29c (Supplementary Fig. S4a, b). We also determined the effect of AC005562.1 on the precursor of hsa-miR-29c (pre-hsa-miR-29c) by using the pre-miRNA assay. Note that the expression of pre-miR-29c was about 3 folder and 6 folder lower in KYSE150 and KYSE410 cells expressing AC005562.1, compared with control cells, respectively (Supplementary Fig. S4c). The copy numbers of AC005562.1 and hsa-miR-29c were analyzed and they are found in the same order of magnitudes (Supplementary Fig. S4d). Our results showed that MS2-tagged AC005562.1 enriched lots of hsa-miR-29c compared with the control, indicating the direct interaction between AC005562.1 and miR-29c (Fig. 1g). Moreover, the luciferase reporter assay revealed that hsa-miR-29c overexpression reduced the luciferase activity in the cells transfected with the plasmid expressing wild-type AC005562.1 but not the mutant plasmid (Supplementary Fig. S4e). More importantly, a pull-down assay showed that AC005562.1 was enriched in the captured fraction in the biotinylated wild-type hsa-miR-29c group, and mutation of the AC005562.1 binding site on hsa-miR-29c disrupted the interaction between AC005562.1 and hsa-miR-29c (Fig. 1h). Furthermore, we noted that overexpression of hsa-miR-29c rescued the enhancing effect of AC005562.1 on cancer cell invasion. In contrast, downregulation of hsa-miR-29c was found to attenuate the inhibitory effect of AC005562.1 knockdown on cell invasion (Supplementary Fig. S4f, g). Taken together, we proposed that AC005562.1 selectively inhibits the maturation process of miR-29c and result in the decreased mature miR-29c.

Subsequently, in silico analysis was carried to search the candidate targets and FSTL1 became the focus of our research (Figs. 1I and Supplementary Fig. S5a). FSTL1 was upregulated in LM3 and I6 cells (Supplementary Fig. S5b), and overexpression of hsa-miR-29c led to decreased FSTL1 expression and inactivation of the downstream NFκB signaling pathway and vise versa (Supplementary Fig. S5c, d). The knockdown of FSTL1 expression or the presence of NFκB signaling pathway inhibitor (BAY11-7082) was found to effectively attenuate the effect of AC005562.1 on the downstream NFκB signaling pathway and cell invasion (Supplementary Fig. S5e, f). The negative regulation of miR-29c on FSTL1 was observed in the cells transfected with hsa-miR-29c mimic or inhibitors (Supplementary Fig. S5g). The direct targeting of FSTL1 by hsa-miR-29c was demonstrated in dual-luciferase reporter assay (Supplementary Fig. S5h), moreover, FSTL1 overexpression abolished the inhibitory effect of hsa-miR-29c on ESCC cell invasion and EMT markers, and vise versa (Supplementary Fig. S5i–k). FSTL1 was upregulated in tumor tissues, and further increased in metastatic tissues, and its expression was negatively correlated with survival of ESCC patients and hsa-miR-29c expression (Supplementary Fig. S5l–o). More importantly, AC005562.1 indeed induced the expression of FSTL1, which was abrogated by hsa-miR-29c (Fig. 1j). Conversely, knockdown of FSTL1 abolished the promoting effect of AC005562.1 on cell invasion, suggesting that FSTL1 is the direct target of hsa-miR-29c and mediates the function of AC005562.1-hsa-miR-29c axis in cancer invasion (Fig. 1k).

The role of AC005562.1-hsa-miR-29c axis in the tumor microenvironment remains to be illustrated. First, a decrease in tube formation was observed in the HUVECs treated with conditioned media (CM) from hsa-miR-29c-expressing ESCC cells (Supplementary Fig. S6a, b). Gain- and loss-of-function experiments showed the negative regulation of TGFβ2 expression by hsa-miR-29c (Supplementary Fig. S6c–f). We observed not only a reduction in microvessel density but also a decrease in TGFβ2 expression, in the tumor xenografts derived from hsa-miR-29c-overexpressing ESCC cells in nude mice (Supplementary Fig. S6g, h). Upregulation of TGFβ2 in primary ESCC and metastatic tumors as well as its correlation with poor survival were illustrated (Supplementary Fig. S6i–k). A significantly negative correlation between hsa-miR-29c and TGFβ2, as well as a positive correlative between AC005562.1 and TGFβ2, was observed in ESCC tissues (Supplementary Fig. S6l). Moreover, TGFβ2 was found to be directly targeted by hsa-miR-29c and mediate the effect of hsa-miR-29c on tube formation (Supplementary Fig. S6m, n). More importantly, AC005562.1 could induce the expression of TGFβ2, whereas the effect was rescued by overexpression of hsa-miR-29c (Fig. 1l). Furthermore, our results demonstrated that overexpression of AC005562.1 promoted tumor angiogenesis in vitro and in vivo, and this effect was significantly abolished by knockdown of TGFβ2 (Fig. 1m and Supplementary Fig. S6o). These results suggest that AC005562.1-hsa-miR-29c axis may exert its effect in the tumor microenvironment by directly targeting TGFβ2.

We also examined the therapeutic efficacy of hsa-miR-29c oligonucleotide in the mouse model. The results showed that hsa-miR-29c treatment led to a significant decrease in lung metastasis, as evidenced by bioluminescence imaging (Supplementary Fig. S7a–c), suggesting that systemic delivery of hsa-miR-29c may suppress tumor metastasis without significant toxic effects (Supplementary Fig. S7e, f).

Taken together, we demonstrate the important role of the AC005562.1-hsa-miR-29c regulatory axis in regulating tumor metastasis and microenvironment, and identify FSTL1 and TGFβ2 as direct downstream targets that mediate the function of the AC005562.1-hsa-miR-29c axis (Fig. 1n). The outcome of this study will facilitate the identification of functional biomarkers in esophageal cancer and provide useful preclinical data for the development of novel systemic therapies for this lethal disease.

## Supplementary information

Supplementary materials
