# Supplementary material for: Genome-wide identification of key regulatory lncRNAs in esophageal cancer metastasis
Source: Signal Transduct Target Ther. 2021 Feb 27;6:88. doi: 10.1038/s41392-021-00476-9 (PMC7910292; doi:10.1038/s41392-021-00476-9)
Supplement: Supplementary file 1 — Supplementary materials [file 41392_2021_476_MOESM1_ESM.docx]

Supplementary Materials for

Genome-wide identification of key regulatory lncRNAs

in esophageal cancer metastasis

Wen Wen Xu^1#^, Can-Can Zheng^2#^, Qian Zuo^2#^, Jun-Qi Li^1^, Pan Hong^2^, Yan-Ru Qin^3^, Xin-Yuan Guan^4^, Qing-Yu He^2^, Hua-Xin Liao^1*^, Bin Li^2*^

Correspondence to: [libinjnu@163.com](mailto:libinjnu@163.com), [tliao805@jnu.edu.cn](mailto:tliao805@jnu.edu.cn)

^1^MOE Key Laboratory of Tumor Molecular Biology and Guangdong Provincial Key Laboratory of Bioengineering Medicine, National Engineering Research Center of Genetic Medicine, Institute of Biomedicine, College of Life Science and Technology, Jinan University, Guangzhou 510632, China.

^2^MOE Key Laboratory of Tumor Molecular Biology and Key Laboratory of Functional Protein Research of Guangdong Higher Education Institutes, Institute of Life and Health Engineering, College of Life Science and Technology, Jinan University, Guangzhou 510632, China;

^3^State Key Laboratory of Esophageal Cancer Prevention and Treatment, Department of Clinical Oncology, First Affiliated Hospital, Zhengzhou University, Zhengzhou, China.

^4^Department of Clinical Oncology, Li Ka Shing Faculty of Medicine, The University of Hong Kong, Pokfulam, Hong Kong SAR, China.

**This PDF file includes:**

Materials and Methods

Supplementary Text

Figures. S1 to S6

Tables S1 to S5

**Materials and Methods**

Cell lines and culture

The human ESCC cell lines KYSE150 and KYSE410 obtained from DSMZ (Braunschweig, Germany) were maintained in RPMI 1640 (Sigma, St Louis, MO, USA) supplemented with 10% fetal bovine serum (FBS) (Invitrogen, Gaithersburg, MD, USA). The 293T cells (ATCC, Manassas, VA, USA) were maintained in DMEM (Sigma) supplemented with 10% FBS. Human umbilical vein endothelial cells (HUVECs) were purchased from Invitrogen.

Plasmids, recombinant proteins, transfection and infection

The overexpressing and -knockdown plasmids of AC005562.1, FSTL1 and TGFβ2 were purchased from TranSheep Bio (Shanghai, China). The expressing plasmids of hsa-miR-29c and the scrambled miRNA control as well as the plasmids expressing miR-zip-29c and miR-zip-CON were constructed. The hsa-miR-29c mimics and corresponding negative controls as well as the miRIDIAN anti-hsa-miR-29c inhibitor and the negative control were purchased from Ambion (Austin, TX, USA). The 3'UTR of FSTL1 or TGFβ2 was introduced into pGL3-control (Fitchburg, WI, USA) to establish the pGL3-FSTL1-3'UTR and pGL3-TGFβ2-3'UTR plasmid, respectively. Recombinant human TGFβ2 were obtained from PeproTech (Rocky Hill, NJ, USA). NFκB signaling pathway inhibitor (BAY11-7082) was purchased from Cell Signaling Technology (Danvers, MA, USA).

LncRNA library preparation and sequencing

The LncRNA library preparation and sequencing was performed by Annoroad Corporation (Beijing, China). In brief, a total amount of 3 μg RNA per sample was used as initial material for the RNA sample preparations. Total RNA was treated to remove ribosomal RNA (rRNA) by using Epicentre Ribo-Zero^TM^ Gold Kits (Human/Mouse/Rat/other) (Epicentre, USA). Subsequently, the sequencing libraries were generated following manufacturer recommendations with varied index label by NEBNext® Ultra™ Directional RNA Library Prep Kit for Illumina (NEB, Ispawich, USA). The clustering of the index-coded samples was performed on a cBot cluster generation system using TruSeq PE Cluster Kit v4-cBot-HS (Illumina) according to the manufacturer’s instructions. After cluster generation, the libraries were sequenced on an Illumina platform and 150 bp paired-end reads were generated.

 TaqMan low density array

TaqMan low density arrays (TaqMan Arrays) were taken to perform relative quantitation (RQ) of targets using the comparative CT (ddCT) method on 7900HT Systems (Applied Biosystems, Carlsbad, CA, USA). In brief, DNA was synthesized from total RNA samples using the High Capacity cDNA Archive Kit (Applied Biosystems). Next, the sample-specific PCR mix was loaded into a TaqMan Array which is pre-loaded with TaqMan® Gene Expression Assays. The TaqMan Array is then run on the 7900HT system for quantitative real-time PCR analysis.

Tissue microarray

A tissue microarray (TMA) containing 104 cases of ESCC tissues and 74 cases of paired normal tissues (Shanghai Outdo Biotech, Shanghai, China) was used to determine the expression patterns of AC005562.1, hsa-miR-29c, FSTL1 and TGFβ2 as well as their correlation with clinicopathological parameters. A TMA consisting of 40 pairs of primary ESCC and matched lymph nodes with metastatic ESCC (US Biomax, Rockville, MD, USA) was included to compare AC005562.1, hsa-miR-29c, FSTL1 and TGFβ2 expression in primary tumors with that in metastasized tumors of ESCC. Another TMA consisting of 17 cases primary ESCC and 22 cases metastatic tissues was taken to investigate the expression of TGFβ2 (Shanghai Outdo Biotech).

In situ hybridization

The tissue microarray was deparafﬁnized in xylene and rehydrated with graded alcohol. Slides were then digested with 8 mg/ml pepsin at 3°C for 10 min, washed, and then dehydrated in graded alcohol. The slides were hybridized with the probes against AC005562.1 (40 nM), hsa-miR-29c (40 nM) and a control probe (Exiqon, Vedbaek, Denmark), respectively, at 60°C for 16 hr. Each TMA core was scored by two pathologists who had no prior knowledge of patient data. The intensity of staining in tumor cells was categorized into four groups: score 0 (negative), score 1 (weakly positive), score 2 (moderately positive), and score 3 (strongly positive).

Immunohistochemistry

Anti-FSTL1 (#AF1694, 1:1000 dilution, R&D Systems, Minneapolis, MN, USA) and TGFβ2 antibody (#19999-1-AP, 1:500 dilution, Proteintech, Rosemont, IL, USA ) was used in this study. The slides were conterstained with hematoxylin and eosin, and the staining intensity was categorized into four scores. The CD31 primary antibody (#sc-1506; 1:100 dilution) was purchased from Santa Cruz Biotechnology (Santa Cruz, CA, USA). Microvessel density was calculated as the mean number of CD31-positive vessels in six random ﬁelds from representative tumor sections.

Chamber invasion assay

In brief, the invasive activity of ESCC cells was evaluated with the use of 8 µm pore-size invasion chamber coated with matrigel (BD Biosciences, Bedford, MA, USA). The cells suspended in serum-free medium were seeded into the upper compartment, and the lower chamber was filled with complete medium. Following the incubation, cells were fixed in 100% methanol for 15 min, stained with 0.2% crystal violet for 5 min, and then counted. The experiment was performed in triplicate.

Tube formation assay

In brief, HUVECs were seeded in a 96-well plate that was pre-coated with matrigel (BD Biosciences). The images of capillary-like tubes were taken, and the total length of tube formation was quantified in 6 random fields from each well.

Cell proliferation assay

Cell viability was measured with the use of a WST-1 Cell Proliferation and Cytotoxicity Assay Kit (Beyotime Biotechnology, Shanghai, China).

Western blot

The primary antibodies used included FSTL1 (#AF1694, 1:1000 dilution) from R&D Systems, E-cadherin (#3195, 1:2000 dilution), N-cadherin (#13116, 1:1000 dilution), Vimentin (#5741, 1:1000 dilution), p-p65 (#3033, 1:500 dilution) and p65 (#8242, 1:1000 dilution) from Cell Signaling Technology, TGFβ2 (#19999-1-AP, 1:2000 dilution) from Proteintech, actin from Santa Cruz Biotechnology (#sc-1616; , 1:3000 dilution, Santa Cruz, CA, USA).

Reverse transcription and quantitative real-time polymerase chain reaction (qRT-PCR)

Total RNA was isolated using Trizol reagent according to the manufacturer’s protocol (Invitrogen). The expression of hsa-miR-29c was also quantified using the TaqMan miRNA assay kit (Applied Biosystems) according to the manufacturer’s instructions. Human small nuclear U6 RNA was included as internal control for miRNA detection.

Quantitative real-time PCR to determine copy number

Total DNA was extracted by using a HiPure Tissue DNA mini Kit (Magen, Guangzhou, China), and qPCR was performed to amplify AC005562.1 and hsa-miR-29c. Absolute quantification was achieved by relating the measured Ct values to a standard curve, which can be obtained by serial dilutions of genome-equivalent amounts of a plasmid containing the gene sequence. The sequences of primers were listed in Supplementary Table S5.

TaqMan pre-miRNA assay

TaqMan pre-miRNA assay kit (Applied Biosystems) was used to quantify precursor miRNAs of has-miR-29c (pre-miR-29c) according to the manufacturer’s instructions. Briefly, total RNA was isolated from ESCC cells with Trizol reagent (Invitrogen). The DNA-free RNA obtained after treating with TURBO DNA-free kit (Ambion, Foster City, CA, USA) was subjected to TaqMan pre-miRNA assay.

Enzyme-linked immunosorbent assay (ELISA)

A Human TGFβ2 ELISA Kit (RayBiotech, Norcross, GA, USA) was used to determine the expression level of TGFβ2 in the conditioned medium according to the manufacturer’s instructions.

MS2-RNA immunoprecipitation (MS2-RIP)

To obtain the miRNAs associated with AC005562.1, the MS2-tagged AC005562.1 plasmid or vector control was transfected into the KYSE150 cells stably expressing MS2-GST fusion protein. After 48 h, the cells were subjected to RIP analysis by using the Magna RIP™ RNA-Binding Protein Immunoprecipitation Kit (Millipore, Bedford, MA, USA). The immunoprecipitated RNA was determined by real-time PCR to confirm the presence of hsa-miR-29c.

Pull-down assay

The biotinylated widetype hsa-miR-29c and mutant miR-29, as well as nonsense control were constructed (GenePharma, Shanghai, China). After transfection for 48 hours, KYSE150 cells were harvested, lysated, sonicated and incubated with M-280 streptavidin beads according to the manufacture’s protocol at 4 °C overnight (Invitrogen). After washing, the bound RNAs were eluted and purified using TRIzol and the abundance of AC005562.1 was determined by qRT-PCR assay.

Site mutation design and luciferase assay

To construct the plasmids used in dual-luciferase reporter assays, fragments of the FSTL1, TGFβ2 3'-UTR and AC005562.1 containing the hsa-miR-29c binding sites and fragment containing mutations of these sites generated by the QuikChange Lightning Site-Directed Mutagenesis Kit (Agilent Technologies, Santa Clara, CA, USA) were ampliﬁed by PCR using specific primers and cloned into the psiCHECK-2 vector (Promega, Madison, WI, USA). After incubation for 48 h, the firefly and Renilla luciferase activities were detected, respectively. Relative luciferase activity was normalized to the firefly luciferase internal control. Sequences of the primers used for generating the mutations are listed in Supplementary Table S3. Luciferase activity was measured using the Dual-Luciferase Reporter Assay (Promega) according to the manufacturer’s instructions.

Quantitative real-time PCR to determine copy number

Total DNA was extracted by using a HiPure Tissue DNA mini Kit (Magen, Guangzhou, China), and qPCR was performed to amplify AC005562.1 and hsa-miR-29c. Absolute quantification was achieved by relating the measured Ct values to a standard curve, which can be obtained by serial dilutions of genome-equivalent amounts of a plasmid containing the gene sequence

Tumor xenograft model

All the animal experiments were approved by the Ethics Committee for Animal experiments of Jinan University. In brief, the ESCC cells KYSE150 expressing hsa-miR-29c or control were subcutaneously injected into the flanks of nude mice and the tumor xenografts were collected for immunohistochemical or Western blot analysis.

Experimental metastasis model and oligo treatment

Briefly, 1×10^6^ luciferase-expressing KYSE150-luc cells were injected intravenously into the tail vein of nude mice. For the treatment experiment, hsa-miR-29c oligonucleotide or miR-CON (GenePharma, Shanghai, China) was formulated with a polymer-based agent (in vivo-jetPEI; Polyplus, Illkirch, France) and injected intravenously into mice. Metastasis was monitored weekly by bioluminescent imaging.

Hematologic analyses

In brief, Alanine aminotransferase (ALT) and aspartate aminotransferase (AST) in mouse serum were determined using commercial kits (HuiLi Biotech Ltd., Changchun, China). Hemoglobin (HGB) and blood cells from mice including lymphocytes, neutrophils, platelets (PLT), red blood cells (RBC) and white blood cells (WBC) were analyzed by a fully automatic hematology analyzer (BC-2800Vet, Mondrary, Shenzhen, China).

Public datasets analysis

The binding between LncRNA and miRNA was analyzed by using public software (LncBase Predicted v.2, experimental module, http://carolina.imis.athenainnovation.gr/diana_tools/web/index.php?r=lncbasev2%2Findex) and RegRNA 2.0 (http://regrna2.mbc.nctu.edu.tw/). The expression of AC005562.1 in The Cancer Genome Atlas (TCGA) datasets were analyzed by using the online softwares TANRIC (https://ibl.mdanderson.org/tanric/_design/basic/index.html). The GEPIA2 (http://gepia2.cancer-pku.cn/#index) was taken to analyze the expression of AC005562.1 in esophageal cancer. Two software programs including TargetScan (http://www.targetscan.org) and miRanda (http://www.microrna.org/micro rna/getExpr Form.do) were used to predict the biological targets of hsa-miR-29c.

Statistical analysis

All *in vitro* experiments and assays were repeated at least 3 times. The data were expressed as mean ± SD and compared by t-test. Survival analysis was performed by Kaplan-Meier method with the log-rank test using the Statistical Package for the Social Sciences (SPSS) (SPSS Inc, Chicago, IL). P values < 0.05 were considered as significant for all experiments.

Supplementary Text

Acknowledgements

This work was supported by National Natural Science Foundation of China (Project Nos. 81672953，81803551, 81773085, 31961160727); Guangdong Innovative and Entrepreneurial Research Team Program (Project No. 2013Y113); Zhuhai Innovative and Entrepreneurial Research Team Program (Project No. ZH01110405160015PWC); National Key R & D Program of China (2017YFA0505100); Guangzhou Science and Technology Project (201904010061); and the Fundamental Research Funds for the Central Universities (21620429).

**Figure. S1**

**
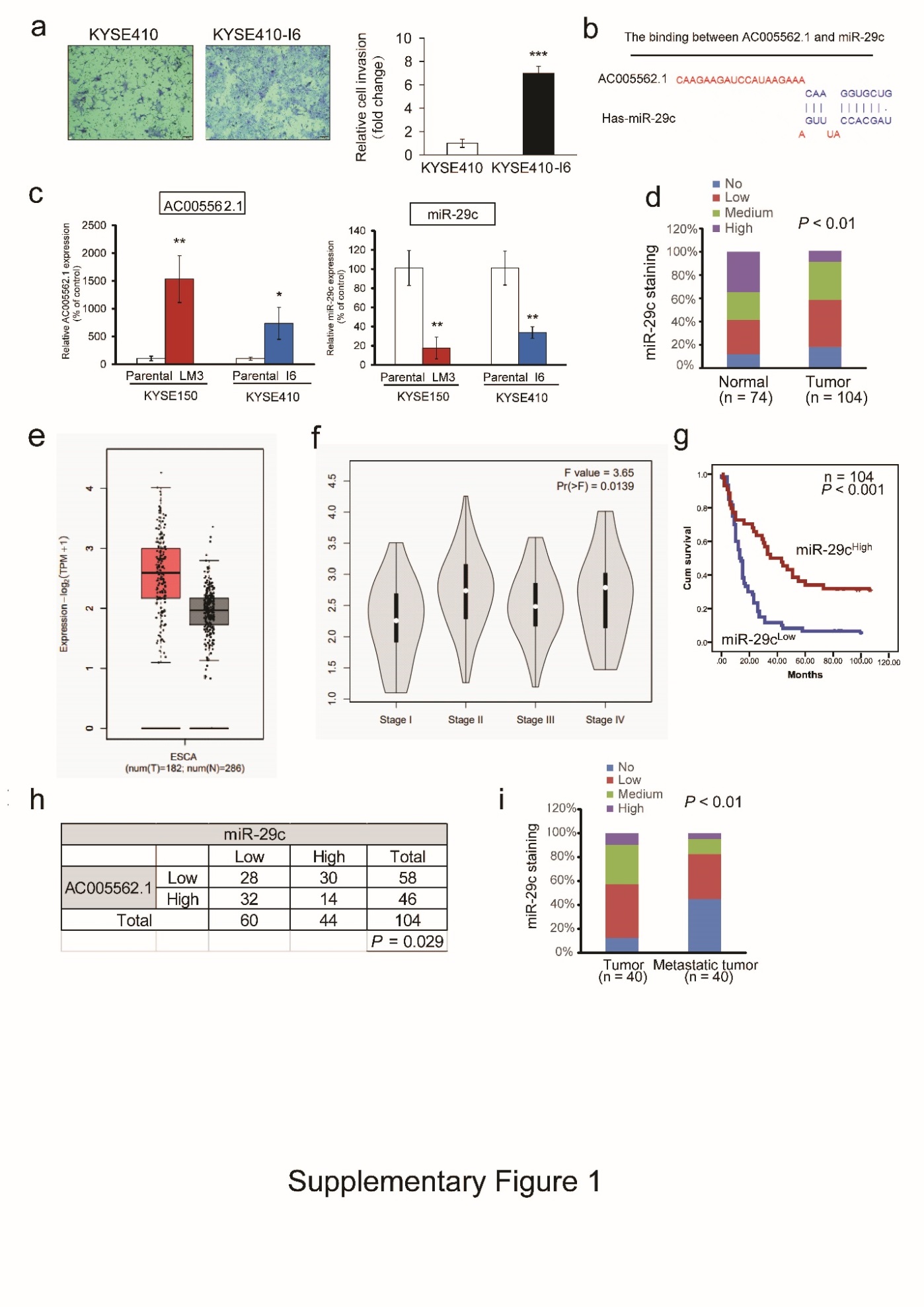
**

**Fig. S1 (a)** Boyden chamber comparing the invasive ability of I6 cells and parental cells. **(b)** The predicted complementary sequence between AC005562.1 and has-hsa-miR-29c. **(c)** qRT-PCR results comparing the expression of AC005526 and hsa-miR-29c in LM3 and I6 cells compared with parental cells, respectively. **(d)** Expression pattern of AC005562.1 and hsa-miR-29c in 74 normal tissues and 104 ESCC tissues. **(e-f)** Expression pattern of AC005562.1 in esophageal cancer by GEPIA2. **(g)** Kaplan-Meier plots were used to compare the overall survival of 104 patients with ESCC stratiﬁed according to AC005562.1 or hsa-miR-29c expression. **(h)** Pearson chi-square analysis showing the correlation between AC005562.1 and hsa-miR-29c. **(i)** Expression pattern of hsa-miR-29c in 40 pairs of primary esophageal cancer and matched metastatic tissues. Bars, SD; *, P < 0.05; **, P < 0.01

**Figure. S2**


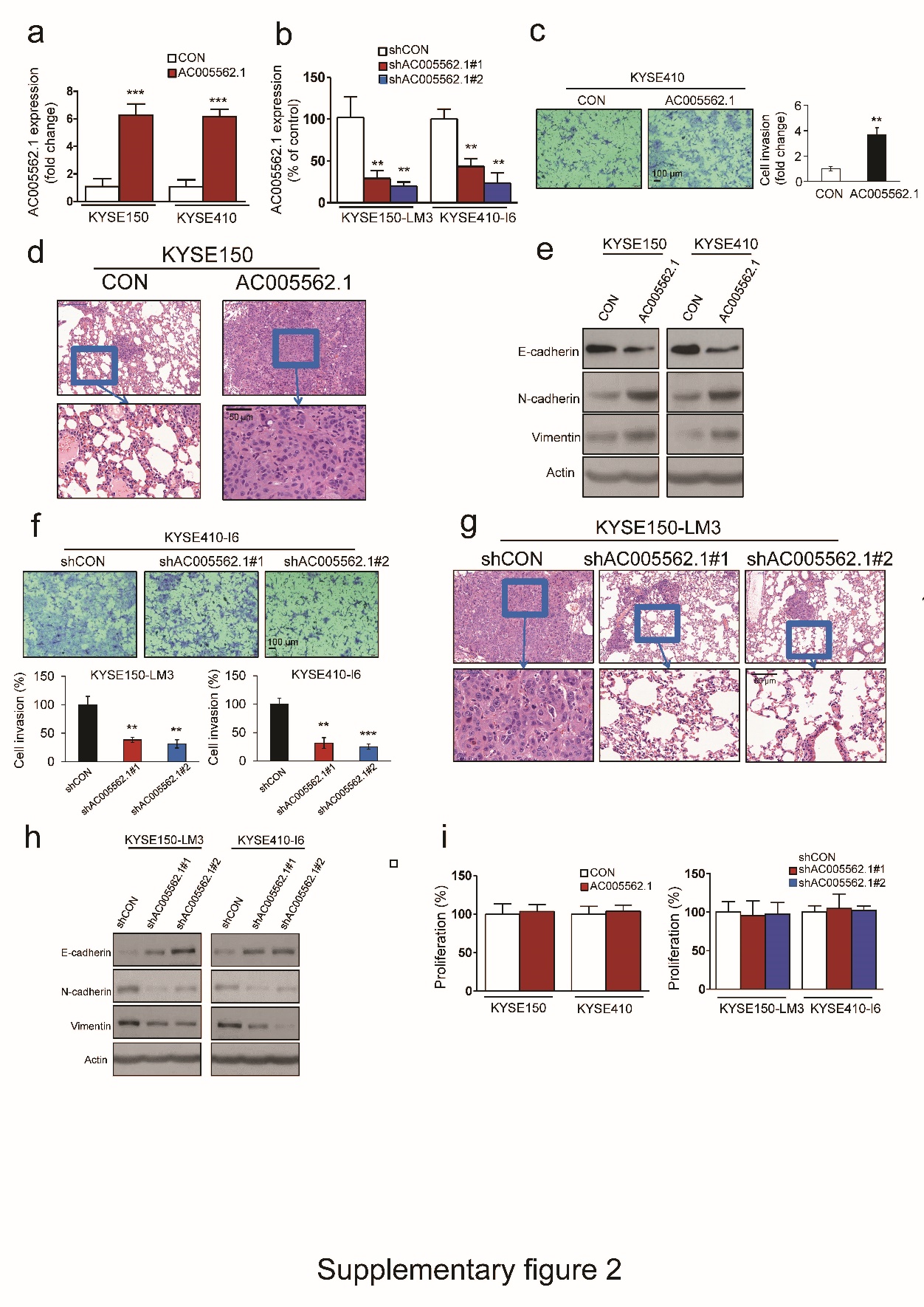


**Fig. S2 (a-b)** RT-PCR showing the expression of AC005562.1 in cells transfected with AC005562.1-overexpressing plasmids or in cells transfected with shRNA against AC005562.1. **(c)** Chamber invasion assay comparing the invasion between AC005562.1-overexpressing cells and vector control cells. **(d)** Histological analysis of mouse lungs by H & E staining. **(e)** Western blot showing the expression of E-cadherin, N-cadherin and vimentin in AC005562.1-overexpressing cells, with actin as an internal control. **(f)** Chamber invasion assay showing the invasion of AC005562.1-knockdown cells compared with the vector control cells. **(g)** Histological analysis of mouse lungs by H & E staining.**(h)** Western blot showing the expression of E-cadherin, N-cadherin and vimentin. **(i)** WST-1 assay showing the effect of AC005562.1 on cell proliferation. Bars, SD; **, P < 0.01; ***, P < 0.001.

**Figure. S3**


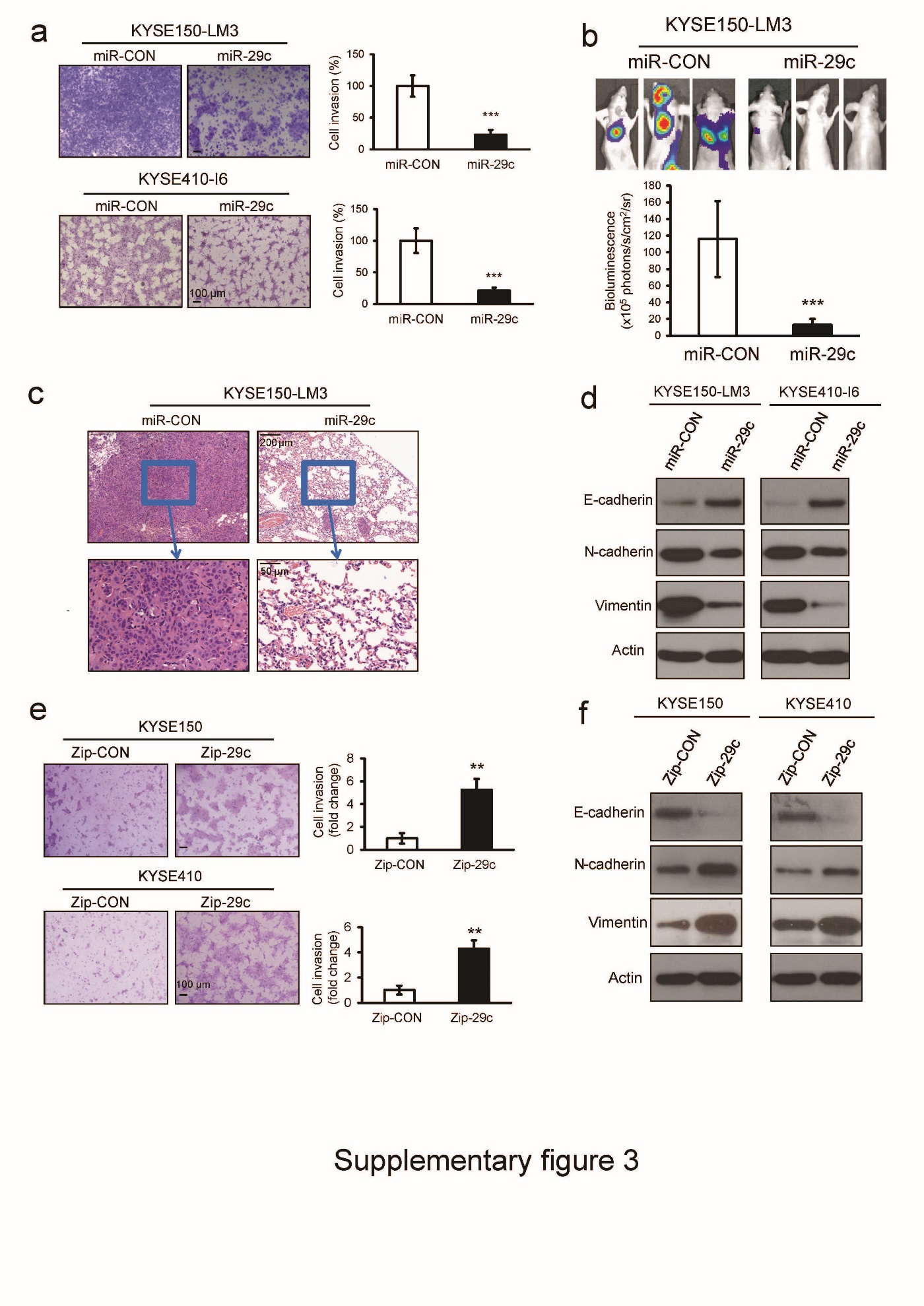


**Fig. S3 (a)** Chamber invasion assay showing invasion of hsa-miR-29c-overexpressing cells. **(b)** Bioluminescent imaging and quantification showing the effect of hsa-miR-29c overexpression on metastasis in an experimental tail vein injection model (n = 6). **(c)** H & E staining of the dissected lungs. **(d)** Western blot showing the expression of E-cadherin, N-cadherin and vimentin in hsa-miR-29c-overexpressing cells, with actin as an internal control. **(e)** Chamber invasion assay showing invasion of hsa-miR-29c-knockdown cells. **(f)** Western blot showing the expression of EMT markers in hsa-miR-29c-knockdown cells. Bars, SD; **, *P* < 0.01; ***, *P* < 0.001.

**Fig. S4**


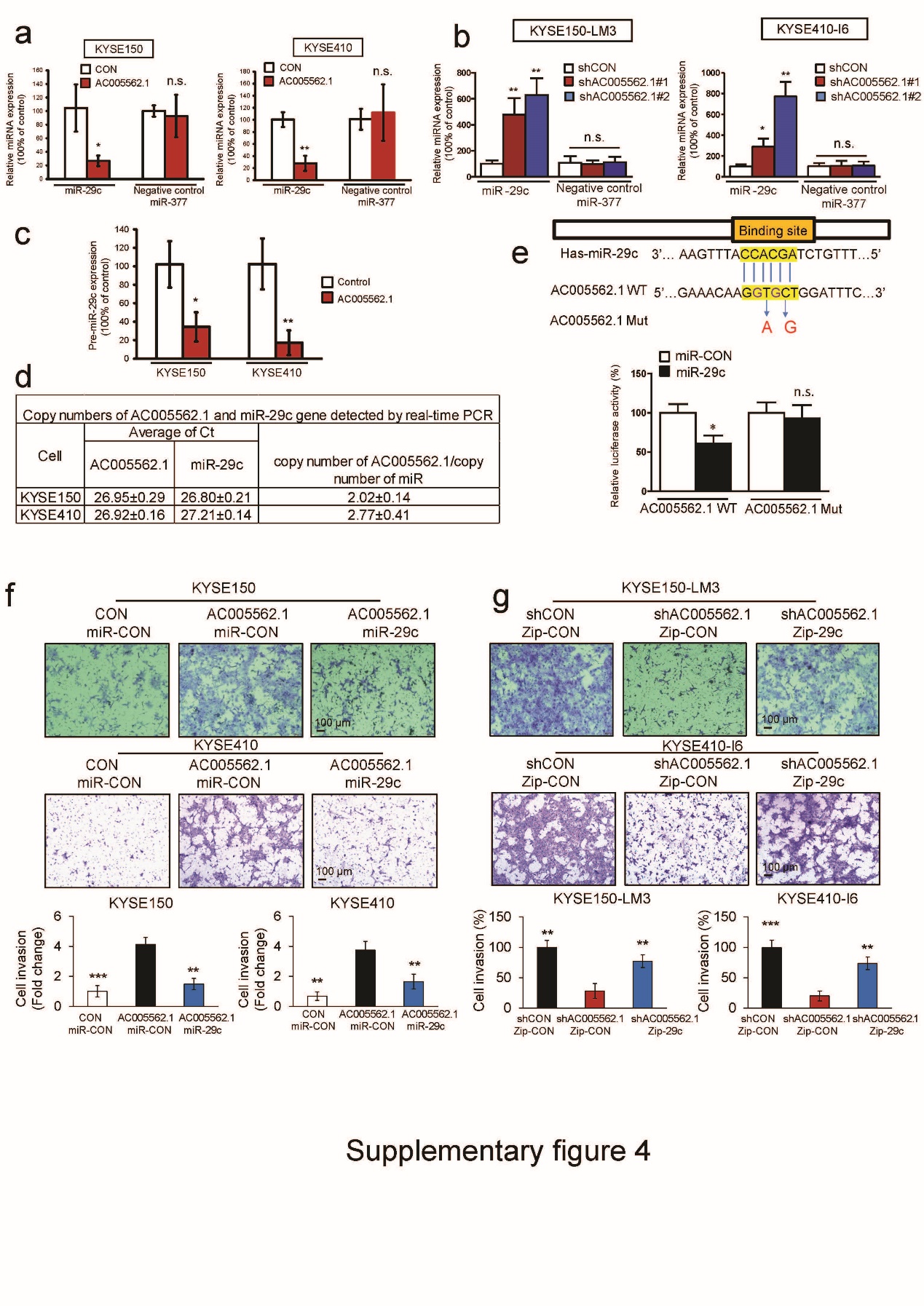


**Fig. S4 (a)** KYSE150 and KYSE410 cells were transfected with AC005562.1 plasmids and then subjected to qRT-PCR for hsa-miR-29c expression, with miR-377 as a negative control. **(b)** qRT-PCR showing the expression of hsa-miR-29c when AC005562.1 was knocked down by shRNA approaches. **(c)** The pre-miRNA assay showing the effect of AC005562.1 on the expression of pre-hsa-miR-29c. **(d)** Table showing the copy number of AC005562.1 and hsa-miR-29c detected by real-time PCR. **(e)** Diagram showing the binding between AC005562.1 and hsa-miR-29c, as well as the mutant design in AC005562.1. Luciferase activity was measured in KYSE150 cells co-transfected with miR-29 as well as the wild type (WT) or mutant (Mut) of AC005562.1. **(f-g)** Boyden chamber showing cell invasion when the expression of AC005562.1 and hsa-miR-29c was altered. Bars, SD; *, P < 0.05; **, P < 0.01; ***, P < 0.001.

**Fig. S5**


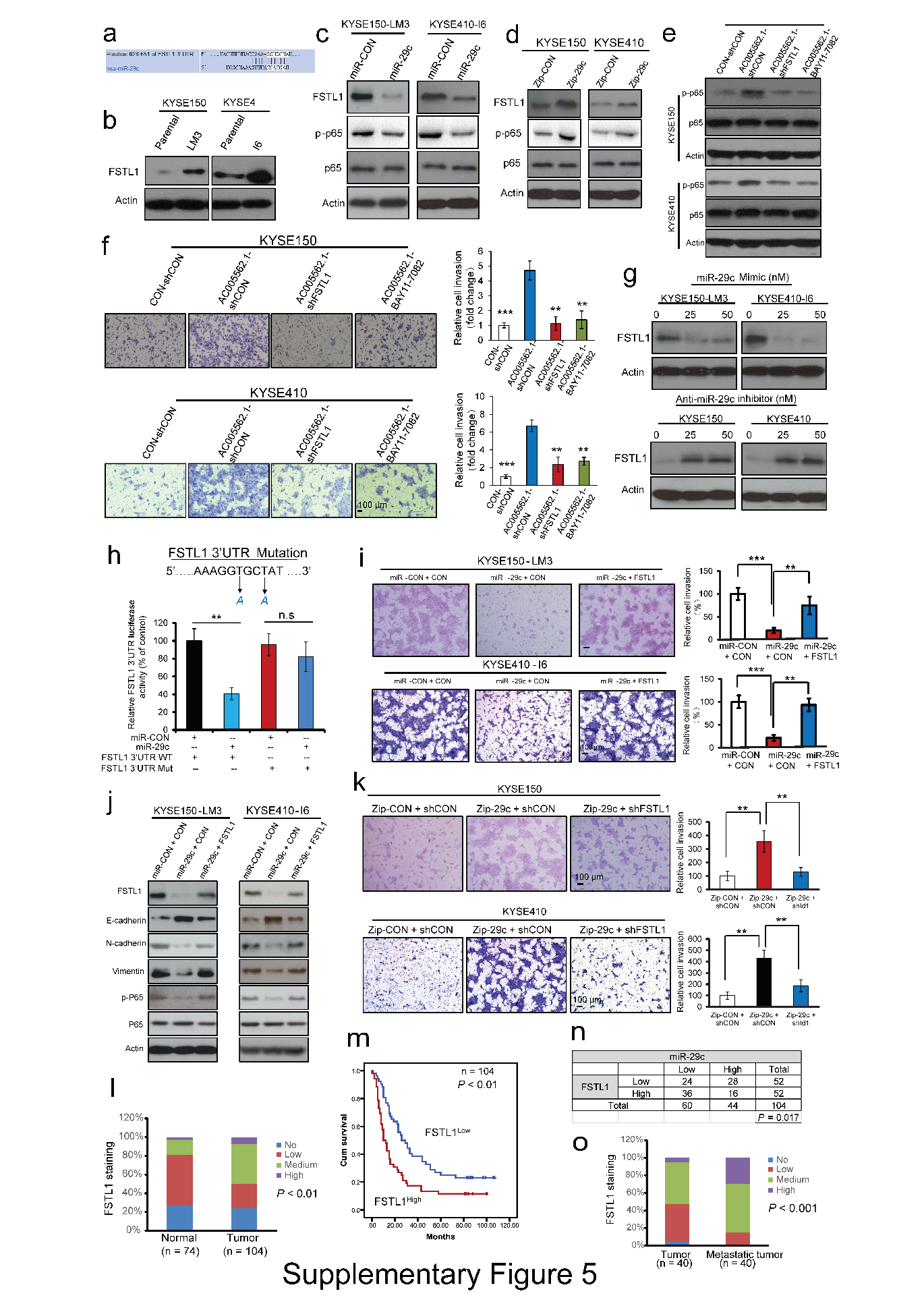


**Fig. S5 (a)** The predicted duplex between hsa-miR-29c and FSTL1 3’UTR is shown. **(b)** Western blot showing the expression of FSTL1 in metastatic cells (LM3) or invasive cells (I6) compared with parental cells. **(c-d)** Western blot showing the expression of FSTL1 and the NFkB signaling pathway when hsa-miR-29c was manipulated. **(e)** Western blot showing the expression of p-p65 when the expression of AC005562.1 and/or FSTL1 was manipulated, in the presence or absence of NFκB signaling pathway inhibitor. **(f)** Boyden chamber assay showing the invasion of cells when the expression of AC005562.1 and/or FSTL1 was manipulated, in the presence or absence of NFκB signaling pathway inhibitor. **(g)** Western blot showing the expression of FSTL1 in the ESCC cells transfected with hsa-miR-29c mimic or inhibitors. **(h)** Mutations in the 3’UTR of FSTL1 (upper panel). Luciferase activity of cells co-transfected with hsa-miR-29c and wild-type or mutant FSTL1 3’UTR. **(i)** Chamber invasion assay showing the invasion of cells overexpressing hsa-miR-29c in the presence or absence of FSTL1. **(j)** Western blot showing the expression of E-cadherin, N-cadherin, vimentin and p-p65 when FSTL1 was altered in the hsa-miR-29c-overexpressing cells. **(k)** Chamber invasion assay showing that knockdown of FSTL1 abolished the effect of hsa-miR-29c knockdown on invasion of ESCC cells. **(l)** Expression of FSTL1 in 74 normal tissues and 104 primary ESCC tissues. **(m)** Kaplan-Meier analysis showing the overall survival of 104 patients with ESCC stratiﬁed according to FSTL1 expression level. **(n)** Pearson chi-square analysis showing the correlation between AC005562.1 and hsa-miR-29c. **(o)** Expression pattern of FSTL1 in 40 paired tumor and metastatic tissues. Bars, SD; **, P < 0.01; ***, P < 0.001.

**Fig. S6**


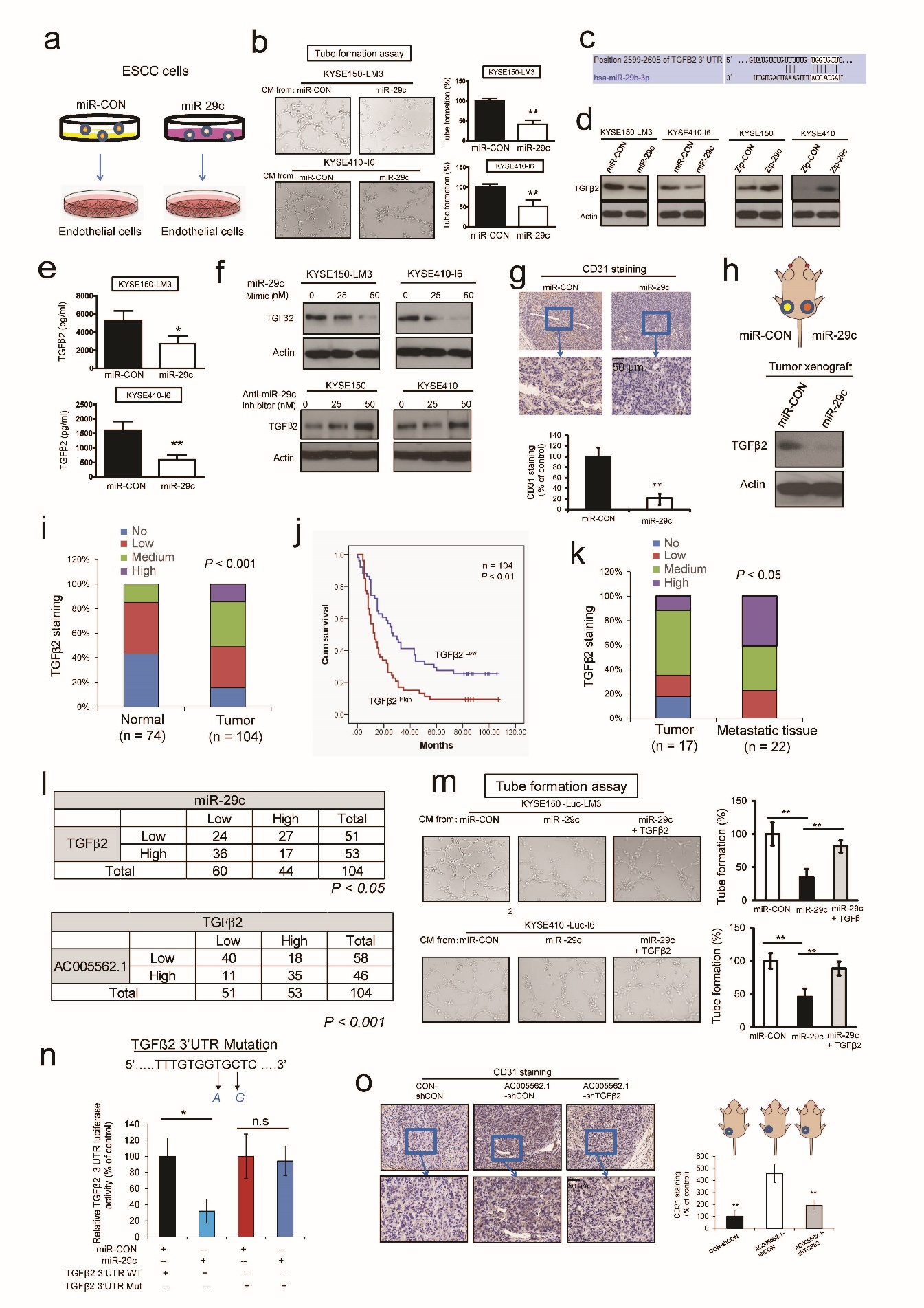


**Fig. S6 (a)** Diagram showing the approach used to collect conditioned medium (CM), then applying the CM to endothelial cells. **(b)** Tube formation assay images and quantification showing the effect of CM from hsa-miR-29c-expressing cells on angiogenesis. **(c)** The complementary sequence between hsa-miR-29c and TGFβ2 by in silico prediction. **(d)** Western blot showing the expression of TGFβ2 protein in the lysates of hsa-miR-29c-expressing ESCC cells. **(e)** ELISA showing the secretion of TGFβ2 in the CM. **(f)** Western blot showing the expression of TGFβ2 in the ESCC cells transfected with hsa-miR-29c mimic or hsa-miR-29c inhibitor. **(g-h)** The microvessel density, indicated by CD31, was montiored (n = 3). **(i)** The expression pattern of TGFβ2 in 74 normal tissues and 104 ESCC tissues. **(j)** Kaplan-Meier plots were used to compare the overall survival according to TGFβ2 expression. **(k)** Expression pattern of TGFβ2 in primary ESCC and metastatic tissues. **(l)** Pearson chi-square analysis showing the correlation between TGFβ2 and hsa-miR-29c, as well as TGFβ2 and AC005562.1. **(m)** Tube formation assay showing that the presence of TGFβ2 recombinant protein rescued the effect of hsa-miR-29c on tube formation. **(n)** Diagram showing the mutations in the 3’-UTR of TGFβ2 (upper panel). Luciferase reporter assay showed that overexpression of hsa-miR-29c decreased the activity of a reporter gene containing wild-type TGFβ2 3’-UTR but not that of a reporter gene containing mutant TGFβ2 3’-UTR. **(o)** AC005562.1 enhanced tumor angiogenesis, indicated by CD31, which was abolished by TGFβ2 knockdown. Bars, SD; *, P < 0.05; **, P < 0.01.

**Fig. S7**


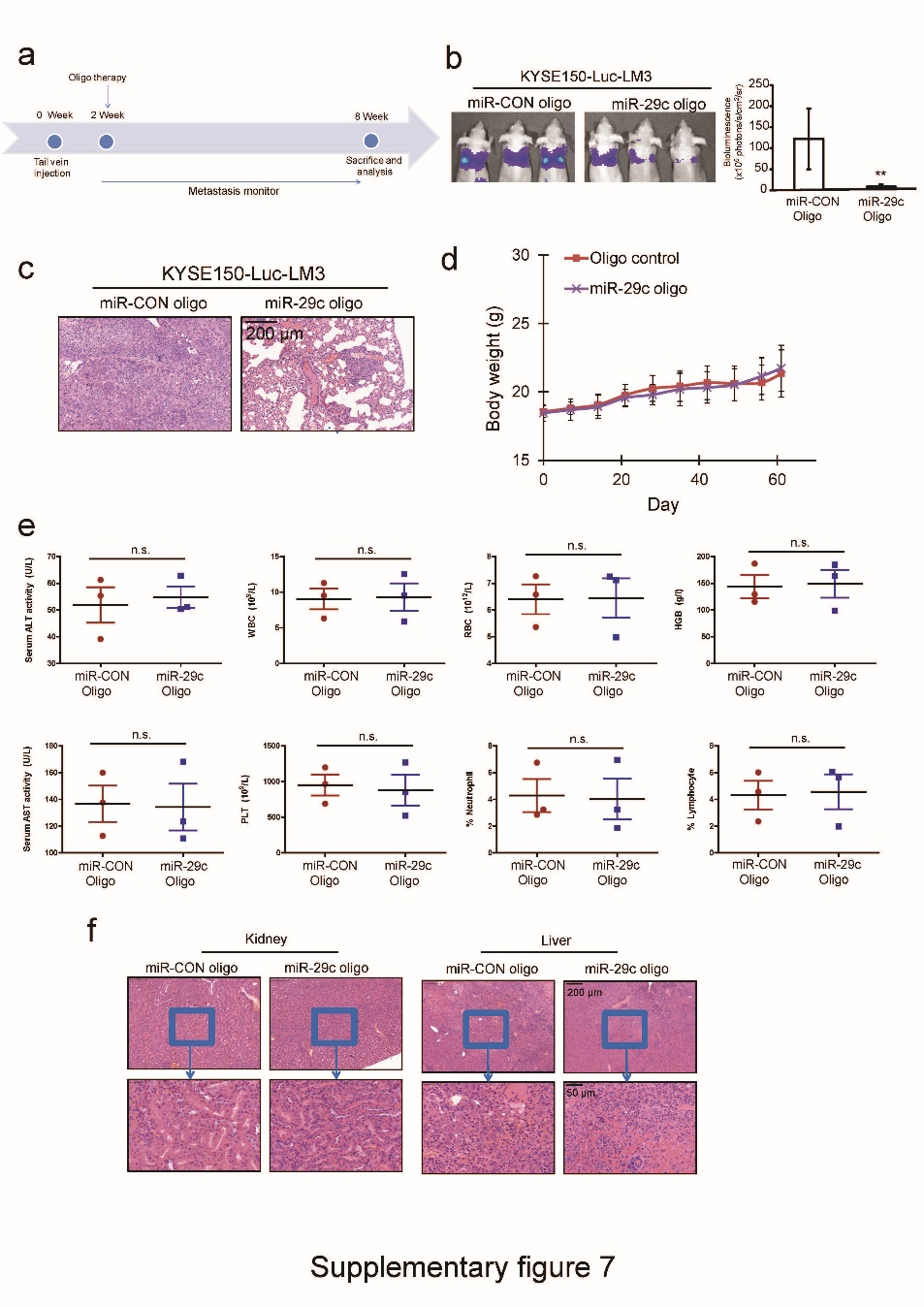


**Fig. S7** **(a)** Experimental setup for *in vivo* treatment. **(b)** Bioluminescence imaging and quantification showing the effect of systemic delivery of hsa-miR-29c on tumor metastasis (n = 6). **(c)** Histological analysis of mouse lungs by H & E staining. **(d)** The body weight of mice was monitored, and there were no significant differences. **(e)** Several indicators were determined in mouse serum, including ALT (alanine aminotransferase), WBC (white blood cell), RBC (red blood cell), HGB (hemoglobin), AST (aspartate aminotransferase), PLT (platelet), neutrophils, and lymphocytes. **(f)** H & E staining of mouse kidneys and liver. Bars, SD; **, P < 0.01.

**Table S1**

The top 150 upregulated lncRNAs in highly metastatic ESCC subline.

|  | Gene name | Fold change |  | Gene name | Fold change |
| --- | --- | --- | --- | --- | --- |
| 1 | RP11-400N13.2 | 514.6 | 46 | RP11-939C17.4 | 12.1 |
| 2 | CTB-105N12.2 | 101.4 | 47 | MIR9-3HG | 12.1 |
| 3 | RP11-867G2.6 | 70.0 | 48 | RP11-43N5.1 | 12.1 |
| 4 | CTC-458G6.4 | 57.9 | 49 | MIR10A | 12.1 |
| 5 | LINC00540 | 41.0 | 50 | RP11-1055B8.3 | 12.1 |
| 6 | RP11-706C16.8 | 41.0 | 51 | AJ003147.8 | 12.1 |
| 7 | RP11-815M8.1 | 38.6 | 52 | RP11-116O18.3 | 12.1 |
| 8 | CTC-490G23.2 | 33.2 | 53 | CTD-3252C9.2 | 12.1 |
| 9 | LINC01504 | 32.0 | 54 | RP11-59H7.4 | 12.1 |
| 10 | PVRL3-AS1 | 29.0 | 55 | RP11-567L7.3 | 12.1 |
| 11 | CTD-2555C10.3 | 27.4 | 56 | AP001962.3 | 12.1 |
| 12 | CTD-2008L17.2 | 26.6 | 57 | CASC15 | 11.3 |
| 13 | AC104076.3 | 24.1 | 58 | AC017074.2 | 10.9 |
| 14 | XXbac-BPG249D20.9 | 21.7 | 59 | AC006262.4 | 10.9 |
| 15 | RP11-867G2.5 | 21.7 | 60 | LINC01537 | 10.3 |
| 16 | RP3-395M20.8 | 19.3 | 61 | MGC16275 | 10.3 |
| 17 | XXbac-BPG55C20.7 | 19.3 | 62 | RP4-781K5.4 | 10.0 |
| 18 | PGM5-AS1 | 19.3 | 63 | LINC00370 | 9.7 |
| 19 | RP11-432J24.3 | 19.3 | 64 | LINC01204 | 9.7 |
| 20 | KB-1507C5.4 | 19.3 | 65 | RP11-702F3.3 | 9.7 |
| 21 | RP11-350J20.12 | 19.3 | 66 | LINC01220 | 9.7 |
| 22 | RP5-963E22.6 | 19.3 | 67 | CTC-497E21.3 | 8.5 |
| 23 | RP11-22P4.2 | 17.5 | 68 | AC011288.2 | 8.5 |
| 24 | RP11-799M12.2 | 16.9 | 69 | RP5-1172N10.2 | 8.5 |
| 25 | CTC-498J12.3 | 16.9 | 70 | RP11-98D18.1 | 8.5 |
| 26 | RP11-553A10.1 | 16.1 | 71 | HDAC11-AS1 | 8.5 |
| 27 | CTD-2066L21.3 | 15.7 | 72 | RP11-707P17.1 | 8.5 |
| 28 | PIK3CD-AS2 | 15.3 | 73 | PCAT19 | 8.5 |
| 29 | AC005562.1 | 14.5 | 74 | RP11-351I21.11 | 8.5 |
| 30 | RP11-1069G10.1 | 14.5 | 75 | CTD-2376I4.1 | 8.5 |
| 31 | RP11-888D10.3 | 14.5 | 76 | RP11-455O6.5 | 8.5 |
| 32 | LRRC2-AS1 | 14.5 | 77 | LINC01219 | 8.1 |
| 33 | RP4-781K5.5 | 13.9 | 78 | LL22NC03-N14H11.1 | 7.5 |
| 34 | ELFN1-AS1 | 13.3 | 79 | LINC00887 | 7.2 |
| 35 | RP11-303E16.6 | 13.3 | 80 | RP11-727F15.13 | 7.2 |
| 36 | AC064834.2 | 12.1 | 81 | AC006460.2 | 7.2 |
| 37 | RP1-159A19.4 | 12.1 | 82 | DPYD-IT1 | 7.2 |
| 38 | RP11-20D14.6 | 12.1 | 83 | FLJ37035 | 7.2 |
| 39 | CYYR1-AS1 | 12.1 | 84 | RP11-191N8.2 | 7.2 |
| 40 | INTS6L-AS1 | 12.1 | 85 | RP11-114M1.1 | 7.2 |
| 41 | AC004603.4 | 12.1 | 86 | AC079305.8 | 7.2 |
| 42 | RP11-10N16.3 | 12.1 | 87 | ZFX-AS1 | 7.2 |
| 43 | CTA-407F11.8 | 12.1 | 88 | RP11-796E2.4 | 7.2 |
| 44 | LINC00322 | 12.1 | 89 | HHIP-AS1 | 7.2 |
| 45 | CTB-127C13.1 | 12.1 | 90 | CTD-2066L21.2 | 7.2 |
|  | Gene name | Fold change |  | Gene name | Fold change |
| 91 | CTB-7E3.1 | 7.2 | 121 | RP11-481J2.2 | 6.0 |
| 92 | RP11-863P13.6 | 7.2 | 122 | RP11-138I1.3 | 6.0 |
| 93 | RP11-104H15.9 | 7.2 | 123 | RP11-660M5.1 | 6.0 |
| 94 | RP11-773H22.4 | 7.2 | 124 | CTA-223H9.9 | 6.0 |
| 95 | CTD-3194G12.2 | 7.2 | 125 | RP11-1399P15.1 | 6.0 |
| 96 | AC009133.21 | 7.2 | 126 | RP11-900F13.2 | 6.0 |
| 97 | bP-21264C1.2 | 7.2 | 127 | AC005593.2 | 6.0 |
| 98 | CTD-2540F13.2 | 7.0 | 128 | RP4-751H13.5 | 6.0 |
| 99 | RP11-7F17.8 | 6.8 | 129 | RP11-989F5.3 | 5.7 |
| 100 | AP001596.6 | 6.6 | 130 | CTD-2357A8.3 | 5.7 |
| 101 | AC073130.3 | 6.6 | 131 | RP11-541P9.3 | 5.6 |
| 102 | CTD-2003C8.2 | 6.6 | 132 | ARHGEF9-IT1 | 5.4 |
| 103 | RP11-649E7.7 | 6.4 | 133 | AC092159.2 | 5.4 |
| 104 | LA16c-425C2.1 | 6.3 | 134 | RP11-98D18.3 | 5.4 |
| 105 | AATBC | 6.3 | 135 | CTD-2320G14.2 | 5.4 |
| 106 | RP11-5L12.1 | 6.2 | 136 | RP11-347C18.3 | 5.4 |
| 107 | RP11-563J2.3 | 6.0 | 137 | LA16c-380H5.1 | 5.3 |
| 108 | MIR3681HG | 6.0 | 138 | RP1-140K8.5 | 5.2 |
| 109 | ZMYND10-AS1 | 6.0 | 139 | CTD-2012K14.8 | 5.2 |
| 110 | RP11-360O19.4 | 6.0 | 140 | RP11-497E19.1 | 5.2 |
| 111 | AF196972.9 | 6.0 | 141 | RP11-463O12.3 | 5.1 |
| 112 | RP11-14I17.2 | 6.0 | 142 | RP11-400K9.4 | 5.1 |
| 113 | RP11-377D9.3 | 6.0 | 143 | RP11-510M2.2 | 5.1 |
| 114 | RP11-972P1.10 | 6.0 | 144 | RP11-635N19.3 | 5.0 |
| 115 | RP11-20I23.5 | 6.0 | 145 | RP13-204A15.5 | 5.0 |
| 116 | AC005082.12 | 6.0 | 146 | AC010127.3 | 5.0 |
| 117 | RP11-94H18.1 | 6.0 | 147 | DUXAP8 | 4.9 |
| 118 | AP000487.6 | 6.0 | 148 | RHOA-IT1 | 4.8 |
| 119 | RP11-54A9.1 | 6.0 | 149 | RP1-153G14.4 | 4.8 |
| 120 | RP11-148B3.1 | 6.0 | 150 | RP11-2H3.6 | 4.8 |

**Table S2**

The top 150 downregulated miRNAs in highly metastatic ESCC subline.

|  | Gene name | Fold change |  | Gene name | Fold change |
| --- | --- | --- | --- | --- | --- |
| 1 | hsa-miR-124 | 0.005 | 46 | hsa-miR-181c | 0.253 |
| 2 | hsa-miR-708 | 0.012 | 47 | hsa-miR-628-5p | 0.26 |
| 3 | hsa-miR-515-3p | 0.032 | 48 | hsa-miR-576-3p | 0.269 |
| 4 | hsa-miR-363 | 0.04 | 49 | hsa-miR-429 | 0.285 |
| 5 | hsa-miR-570 | 0.04 | 50 | hsa-miR-629 | 0.285 |
| 6 | hsa-miR-296-5p | 0.041 | 51 | hsa-miR-190 | 0.292 |
| 7 | hsa-miR-451 | 0.041 | 52 | hsa-miR-361-5p | 0.295 |
| 8 | hsa-miR-208 | 0.042 | 53 | hsa-miR-422a | 0.302 |
| 9 | hsa-miR-331-5p | 0.042 | 54 | hsa-miR-526b | 0.305 |
| 10 | hsa-miR-509-5p | 0.042 | 55 | hsa-miR-128 | 0.311 |
| 11 | hsa-miR-672 | 0.045 | 56 | hsa-miR-130b | 0.311 |
| 12 | hsa-miR-548d-5p | 0.077 | 57 | hsa-miR-339-5p | 0.329 |
| 13 | hsa-let-7c | 0.079 | 58 | hsa-miR-99a | 0.339 |
| 14 | hsa-miR-518f | 0.079 | 59 | hsa-miR-671-3p | 0.341 |
| 15 | hsa-miR-627 | 0.079 | 60 | hsa-miR-19a | 0.342 |
| 16 | hsa-miR-33b | 0.081 | 61 | hsa-miR-98 | 0.345 |
| 17 | hsa-miR-545 | 0.081 | 62 | hsa-miR-135b | 0.349 |
| 18 | hsa-miR-891a | 0.081 | 63 | hsa-miR-10a | 0.352 |
| 19 | hsa-miR-616 | 0.082 | 64 | hsa-miR-28-5p | 0.36 |
| 20 | hsa-miR-299-3p | 0.083 | 65 | hsa-miR-19b | 0.367 |
| 21 | hsa-miR-483-5p | 0.083 | 66 | hsa-miR-339-3p | 0.367 |
| 22 | hsa-miR-495 | 0.083 | 67 | hsa-miR-148a | 0.373 |
| 23 | hsa-miR-367 | 0.084 | 68 | hsa-miR-27a | 0.374 |
| 24 | hsa-miR-376c | 0.084 | 69 | hsa-miR-328 | 0.378 |
| 25 | hsa-miR-125a-3p | 0.086 | 70 | hsa-miR-140-3p | 0.384 |
| 26 | hsa-miR-138 | 0.086 | 71 | hsa-miR-125b | 0.387 |
| 27 | hsa-miR-208b | 0.086 | 72 | hsa-let-7a | 0.393 |
| 28 | hsa-miR-184 | 0.087 | 73 | hsa-miR-20a | 0.395 |
| 29 | hsa-miR-582-5p | 0.094 | 74 | hsa-miR-494 | 0.395 |
| 30 | hsa-miR-369-3p | 0.098 | 75 | hsa-miR-28-3p | 0.402 |
| 31 | hsa-miR-323-3p | 0.134 | 76 | hsa-miR-345 | 0.405 |
| 32 | hsa-miR-34a | 0.145 | 77 | hsa-miR-372 | 0.41 |
| 33 | hsa-miR-193a-5p | 0.162 | 78 | hsa-miR-590-5p | 0.415 |
| 34 | hsa-miR-515-5p | 0.162 | 79 | hsa-miR-126 | 0.42 |
| 35 | hsa-miR-487a | 0.164 | 80 | hsa-miR-652 | 0.421 |
| 36 | hsa-miR-139-5p | 0.171 | 81 | hsa-miR-100 | 0.423 |
| 37 | hsa-miR-449a | 0.184 | 82 | hsa-miR-148b | 0.424 |
| 38 | hsa-miR-489 | 0.189 | 83 | hsa-miR-362-5p | 0.424 |
| 39 | hsa-miR-212 | 0.195 | 84 | hsa-miR-106a | 0.439 |
| 40 | hsa-miR-194 | 0.218 | 85 | RNU48-4373383 | 0.44 |
| 41 | hsa-miR-20b | 0.224 | 86 | hsa-miR-152 | 0.448 |
| 42 | hsa-miR-96 | 0.241 | 87 | hsa-miR-449b | 0.462 |
| 43 | RNU44-4373384 | 0.245 | 88 | hsa-miR-23b | 0.465 |
| 44 | hsa-miR-424 | 0.247 | 89 | hsa-miR-22 | 0.466 |
| 45 | hsa-miR-381 | 0.25 | 90 | hsa-miR-324-5p | 0.47 |
|  | Gene name | Fold change |  | Gene name | Fold change |
| 91 | hsa-miR-210 | 0.472 | 121 | hsa-miR-192 | 0.588 |
| 92 | hsa-miR-95 | 0.473 | 122 | hsa-miR-454 | 0.59 |
| 93 | hsa-miR-93 | 0.476 | 123 | hsa-hsa-miR-29c | 0.592 |
| 94 | hsa-miR-92a | 0.477 | 124 | hsa-miR-186 | 0.593 |
| 95 | hsa-let-7e | 0.48 | 125 | hsa-miR-149 | 0.608 |
| 96 | hsa-miR-25 | 0.48 | 126 | hsa-miR-203 | 0.608 |
| 97 | hsa-miR-103 | 0.482 | 127 | hsa-miR-218 | 0.613 |
| 98 | hsa-let-7d | 0.494 | 128 | hsa-miR-618 | 0.615 |
| 99 | hsa-miR-132 | 0.498 | 129 | hsa-miR-202 | 0.62 |
| 100 | hsa-miR-106b | 0.502 | 130 | hsa-miR-18b | 0.623 |
| 101 | hsa-miR-26b | 0.503 | 131 | hsa-miR-598 | 0.623 |
| 102 | hsa-miR-185 | 0.505 | 132 | hsa-miR-324-3p | 0.625 |
| 103 | hsa-miR-340 | 0.505 | 133 | hsa-miR-660 | 0.625 |
| 104 | hsa-miR-21 | 0.506 | 134 | hsa-miR-27b | 0.626 |
| 105 | hsa-miR-423-5p | 0.509 | 135 | hsa-miR-23a | 0.634 |
| 106 | hsa-miR-484 | 0.51 | 136 | hsa-miR-30b | 0.641 |
| 107 | hsa-miR-29b | 0.511 | 137 | hsa-miR-532-3p | 0.647 |
| 108 | hsa-miR-140-5p | 0.512 | 138 | hsa-miR-542-3p | 0.648 |
| 109 | hsa-miR-502-3p | 0.523 | 139 | hsa-miR-224 | 0.659 |
| 110 | hsa-miR-24 | 0.526 | 140 | hsa-miR-301b | 0.66 |
| 111 | hsa-miR-15a | 0.527 | 141 | hsa-miR-135a | 0.662 |
| 112 | hsa-miR-342-3p | 0.532 | 142 | hsa-miR-200c | 0.662 |
| 113 | hsa-miR-505 | 0.532 | 143 | hsa-miR-193b | 0.663 |
| 114 | hsa-miR-18a | 0.537 | 144 | hsa-miR-200b | 0.671 |
| 115 | hsa-miR-365 | 0.543 | 145 | hsa-miR-30c | 0.675 |
| 116 | hsa-miR-26a | 0.566 | 146 | hsa-miR-491-5p | 0.675 |
| 117 | hsa-let-7g | 0.572 | 147 | hsa-miR-105 | 0.677 |
| 118 | hsa-miR-17 | 0.572 | 148 | hsa-miR-183 | 0.678 |
| 119 | hsa-miR-492 | 0.576 | 149 | hsa-miR-374b | 0.68 |
| 120 | hsa-miR-744 | 0.582 | 150 | hsa-miR-15b | 0.682 |

Table S3

|  |  | |  |  |
| --- | --- | --- | --- | --- |
| Variable | n | Low AC005562.1 | High AC005562.1 | *P* value |
| Age ( years) |  |  |  |  |
| ≤55 | 20 | 11 | 9 |  |
| >55 | 84 | 47 | 37 | 1.000 |
|  |  |  |  |  |
| Gender |  |  |  |  |
| Female | 28 | 18 | 10 |  |
| Male | 76 | 40 | 36 | 0.288 |
|  |  |  |  |  |
|  |  |  |  |  |
| T-Stage |  |  |  |  |
| 1/2 | 16 | 10 | 6 |  |
| 3/4 | 86 | 47 | 39 | 0.561 |
|  |  |  |  |  |
| N-Stage |  |  |  |  |
| N0 | 47 | 34 | 13 |  |
| N1 | 56 | 23 | 33 | **0.001**** |
|  |  |  |  |  |
| M-Stage |  |  |  |  |
| M1 | 0 | 0 | 0 |  |
| M0 | 104 | 58 | 46 | 1.000 |
|  |  |  |  |  |
| Grade |  |  |  |  |
| I & II | 77 | 42 | 35 |  |
| III & IV | 28 | 17 | 11 | 0.573 |
|  |  |  |  |  |
| Stage |  |  |  |  |
| 1 & 2 | 48 | 35 | 13 |  |
| 3 & 4 | 52 | 20 | 32 | **0.0005***** |
|  |  |  |  |  |

Correlation between AC005562.1 expression levels and clinicopathological parameters in 104 cases of esophageal cancer.

Table S4

|  |  | |  |  |
| --- | --- | --- | --- | --- |
| Variable | n | Low hsa-miR-29c | High hsa-miR-29c | *P* value |
| Age ( years) |  |  |  |  |
| ≤55 | 20 | 10 | 10 |  |
| >55 | 84 | 50 | 34 | 0.438 |
|  |  |  |  |  |
| Gender |  |  |  |  |
| Female | 28 | 13 | 15 |  |
| Male | 76 | 47 | 29 | 0.143 |
|  |  |  |  |  |
|  |  |  |  |  |
| T-Stage |  |  |  |  |
| 1/2 | 16 | 7 | 9 |  |
| 3/4 | 86 | 54 | 32 | 0.158 |
|  |  |  |  |  |
| N-Stage |  |  |  |  |
| N0 | 47 | 21 | 26 |  |
| N1 | 56 | 38 | 18 | **0.017*** |
|  |  |  |  |  |
| M-Stage |  |  |  |  |
| M1 | 0 | 0 | 0 |  |
| M0 | 104 | 60 | 44 | 1.00 |
|  |  |  |  |  |
| Grade |  |  |  |  |
| I & II | 77 | 42 | 35 |  |
| III & IV | 28 | 19 | 9 | 0.221 |
|  |  |  |  |  |
| Stage |  |  |  |  |
| 1 & 2 | 48 | 22 | 26 |  |
| 3 & 4 | 52 | 37 | 15 | **0.010*** |
|  |  |  |  |  |

Correlation between hsa-miR-29c expression levels and clinicopathological parameters in 104 cases of esophageal cancer.

Table S5

The mutation primer list.

| AC005562.1 | F | 5’-tggtcgtcatggaccggaag-3’ |
| --- | --- | --- |
|  | R | 5’-cttgcgagccaaaagtcctc-3’ |
| hsa-miR-29c | F | 5’-cccctacatcataaccgatttcaa-3’ |
|  | R | 5’-tacacaggctgaccgatttct-3’ |
| hsa-miR-29c Mut for AC005562 | F | 5'-gatgtagtattggctaaagtttaccaggttctgtttttgtctgagacttg-3' |
|  | R | 5'-caagtctcagacaaaaacagaacctggtaaactttagccaatactacatc-3' |
| AC005562 Mut for hsa-miR-29c | F | 5'-gaacacaacggagaaatccacctccttgtttcttatggatctt-3' |
|  | R | 5'-aagatccataagaaacaaggaggtggatttctccgttgtgttc-3' |
| FSTL1 3’UTR mutation | F | 5'-ccaaattttaaaaatactttttaccaaaggagcaatttctctgtaaaacacttttttttggcaa-3' |
|  | R | 5'-ttgccaaaaaaaagtgttttacagagaaattgctcctttggtaaaaagtatttttaaaatttgg-3' |
| TGFß2 3’UTR mutation | F | 5'-cacgatgtatgtctgtttttgtggaggtctagtggtaaataaattatttcg-3' |
|  | R | 5'-cgaaataatttatttaccactagacctccacaaaaacagacatacatcgtg-3' |
